# Adapted physical activity in subjects and athletes recovering from covid-19: a position statement of the Società Italiana Scienze Motorie e Sportive

**DOI:** 10.1007/s11332-022-00951-y

**Published:** 2022-05-15

**Authors:** Massimo Venturelli, Annamaria Mancini, Alessandra Di Cagno, Giovanni Fiorilli, Mara Paneroni, Federico Roggio, Giuseppe Musumeci, Pasqualina Buono, Federico Schena, Antonio Paoli

**Affiliations:** 1 Department of Neurosciences, Biomedicine and Movement Sciences, University of Verona, Verona, Italy; 2 Department of Internal Medicine, University of Utah, Salt Lake, USA; 3 Department of Movement Sciences and Wellness, University Parthenope, Naples, Italy; 4 CEINGE-Biotecnologie Avanzate, Napoli, Italy; 5 Department of Movement, Human and Health Sciences, Italian University of Sport and Movement of Rome “Foro Italico”, Rome, Italy; 6 Department of Medicine and Health Sciences, University of Molise, v. De Sanctis 1, 86100 Campobasso, Italy; 7 Respiratory Rehabilitation, Istituti Clinici Scientifici Maugeri IRCCS, Lumezzane, Brescia, Italy; 8 Department of Biomedical and Biotechnological Sciences Anatomy, Histology and Movement Sciences Section, School of Medicine, University of Catania, Via S. Sofia 87, 95123 Catania, Italy; 9 Research Center on Motor Activities (CRAM), University of Catania, 95123 Catania, Italy; 10 Department of Biology, College of Science and Technology, Temple University, Philadelphia, PA 19122 USA; 11 Department of Biomedical Sciences, University of Padova, Padua, Italy

**Keywords:** COVID-19, Adapted physical activity, Muscle function, Cardiovascular function, Cognitive function

## Abstract

Coronavirus disease 2019 (COVID-19) is a worldwide pandemic illness that is impacting the cardiovascular, pulmonary, musculoskeletal, and cognitive function of a large spectrum of the worldwide population. The available pharmacological countermeasures of these long-term effects of COVID-19 are minimal, while myriads of non-specific non-pharmacological treatments are emerging in the literature. In this complicated scenario, particular emphasis should be dedicated to specific exercise interventions tailored for subjects and athletes recovering from COVID-19. Specific guidelines on adapted physical activity in this critical population are unavailable so far, therefore, in this position statement of the Società Italiana di Scienze Motorie e Sportive (SISMeS) the members of the steering committee of the research group Attività Motoria Adattata, Alimentazione, Salute e Fitness have indicated the adapted physical activity approaches to counteract the long-term effects of the COVID-19, both in good health people and athletes.

## Introduction

In December 2019 an infectious respiratory disease, called COVID-19, emerged in Wuhan, China's Hubei province [1–3]. A new coronavirus, named Severe Acute Respiratory Syndrome-coronavirus 2 (SARS-CoV-2), has been isolated by bronchoalveolar lavage fluid from Wuhan COVID-19 patients [4]. Despite the source of SARS-CoV-2 is uncertain, available evidence suggests that it has an animal origin and, most likely, its ecological reservoir resides in bats. SARS-CoV-2 is a member of β-CoVs, like SARS-Cov and Middle East Respiratory Syndrome (MERS), a family of enveloped, positive-sense single-stranded RNA viruses and can give rise to respiratory, hepatic, enteric, and neurological diseases [5, 6]. SARS-CoV-2 infection spread rapidly from China all over the world, so much, so that on March 11, 2020, the World Health Organization (WHO) classified that infection as a pandemic. As a matter of fact, to date 216,303,376 confirmed Coronavirus cases were globally reported, including 4,498,451 deaths in more than 200 countries, areas or territories. (https://covid19.who.int/ (data updated to 30 August 2021).

SARS-CoV-2 is principally transmitted via respiratory droplet in a direct or indirect manner. Primary viral replication happens in the mucosal epithelium of the nasal cavity and pharynx and then spreads in the lower airways and in the gastrointestinal mucosa [7, 8].

COVID-19 patients show some common symptoms at the onset of the disease: fever, cough, myalgia, fatigue or shortness of breath. Subsequently, dyspnea can progressively evolve into acute respiratory distress syndrome (ARDS) or multi-organ failure (MOF) [8]. A major cause of ARDS and multi-organ failure seems to be a cytokine storm that is usually associated with the worsening of many infectious diseases [9, 10]. A cytokine storm also emerged in critically COVID-19 patients, suggesting an association between this event and the severity of the disease [10].

As well as, the perturbation in the balance between the Ang1-7/MasR and AngII/AT1R axes seems to be a basis of the development of Cardiovascular Diseases and renal diseases [11]. The Coronavirus spike protein (S)/ACE2 interaction is crucial for the SARS-CoV infections and after this interaction with SARS-CoV-2, ACE-2 is downregulated and, consequently, the AngII/AT1R axis is favored at the expense of Ang1-7/MasR axis, thus promoting the onset of the respiratory syndrome [12–15]. Some studies, performed on animal models of physical training, supported the theory that exercise can activate Ang1-7/MasR axis inhibiting AngII/AT1R pathway, with cardiovascular beneficial effects [11].

During the emergency plan and before the availability of COVID-19 vaccines, many countries adopted the lockdown policy to prevent of spread and the rate of evolution of the new variants of SARS-CoV-2 As a direct consequence, many people, forced to stay at home for several weeks or months, reported important repercussions on physical and psychological health. In particular, during the lockdown there was a massive reduction in physical activity levels, with substantial loss of muscle mass (from 6% after 10 days to 10% after 30 days), reduction of muscle insulin sensitivity, reduction in cardiorespiratory fitness and alteration of the oxidative metabolism of the skeletal muscle [16]. Furthermore, it has been well described that physical inactivity negatively influences metabolic health, accelerates cognitive decline and increases the risk of death from all causes. This condition was exacerbated in COVID-19 patients who survived the disease and were bedridden [16]. To date, it is known that regular physical activity and routine exercise can counteract and reduce the deleterious effects of inactivity [15]. The American College of Sports Medicine (ACSM) recommends that people in good health engage in moderate physical activity for at least 150 min per week up to 300 min per week [17]. However, the dose–response relationship of the exercise in the post lockdown is currently poorly understood. Moreover, for subjects who have contracted COVID-19, resuming physical activity could be more complicated due to the sequelae of the diseases. In particular, athletes could have a safe post COVID-19 activity resumption, screening for occult myocardial injury and inflammation to exclude subclinical diseases. Therefore, in these cases it is recommended, after the evaluation by the specialist, to establish adapted physical activity programs that can integrate physical rehabilitation programs [18].

In this position statement of the Società Italiana di Scienze Motorie e Sportive (SISMeS) the members of the steering committee of the research group Attività Motoria Adattata, Alimentazione, Salute FITNESS have indicated the adapted physical activity approaches to counteract the long-term effects of the COVID-19, both in general population and athletes (Table 1).

**Table 1:** Overview guidelines for each section approach

COVID-19 adverse effects	Type of exercise	Countermeasures
Pathophysiological mechanism leading to pulmonary impairment in gas exchanges, direct myocardial injury, arrhythmias, and thromboembolism	Endurance training	High-intensity interval training to ameliorate muscular stimulus, reducing cardio-pulmonary involvement. Small muscle mass exercises to decrease exercise intolerance
Skeletal muscle loss due to inactivity and "cytokine storm" with an imbalance between muscle protein synthesis and protein breakdown	Resistance training	Heavy-load contractions (70–85% 1 RM) with medium–low repetitions (6–12) to optimize muscle hypertrophy and implement muscle strength
Distress and psychological alterations due to pandemic confinement trigger anxiety and depressive disorders	Adapted physical activity	Whole-body vibration exercise and mobilization, respiratory training, and chest relaxation during the early stages of acute phases to retrieve better self-esteem
Elite athletes COVID-19 confirmed suspended from competitive sports	Home-based activity	Low-intensity indoor training to maintain cardiovascular and strength endurance and reduce psychological distress

A general overview concerning the different approaches to COVID-19 adverse effects based on endurance training, resistance training, adapted physical activity and home-based activity for those home-restricted

**Table 2:** Endurance training programs for patients recovering from COVID-19 infection

	Fitt	Progression
Interval training (first choice, first period)	F: 3sessions/ week or every day I: AP: reaching BORG scale = 5–6 or 70–80% HR max prd; PP: reaching BORG scale = 2–3 Ti: Session duration from 20 to 40 min AP/PP duration: from 1 to 4 min AP/PP ratio: 1:1 T: Interval training	If * BORG scale < 4; SpO_2_ > 93% and HR < 70% HR max prd you can modify (increase) one of this aspect (1) Duration AP/PP (2) Intensity (3) Duration of session (4) Number of sessions/week
Continuous training	F: 3sessions/ week or every day I: reaching BORG scale = 3–4 or 60–70% HR max prd Ti: Session duration from 20 to 40 min T: Continuous training	If * BORG scale < 4, SpO2 > 93% and HR < 70% HR max prd you can modify (increase) one of this aspect (1) Intensity (2) Duration of session (3) Number of sessions/week

*F*   frequency; *I*   intensity, *Ti*  time,;*T*  type of program; *AP*  active phase; *PP*  passive phase; *SpO*_*2*_  oxygen saturation; *HR*   heart rate, *max*  maximal, *prd*   predicted

*Evaluation to do at the end of each session

## Endurance training programs for patients recovering from COVID-19 infection

As previously mentioned in this manuscript, subjects recovering from COVID-19 pneumonia are encouraged to participate in exercise training programs to reduce the long-term cardiovascular effects of the virus and counteract the sedentary behaviors associated with the lockdown [19]. From a general perspective, exercise-induced ameliorations are generally accompanied by improvements in central and peripheral hemodynamic factors [20, 21]. Additional benefits are the improvement of maximal aerobic capacity ($\dot{\mathrm{V}}{\mathrm{O}}_{2\mathrm{max}}$), that is highly correlated with longevity and independence of the aged population [22, 23], that are the population more impacted by the COVID-19. Indeed, the pathophysiological scenario of patients recovering from the COVID-19 is similar, at least in part, to what has been observed in patients with pulmonary and cardiovascular diseases such as chronic obstructive pulmonary disease (COPD), heart failure (HF) and, in part, with hypertension. In post-COVID-19 patients, pathophysiological mechanism that could primarily lead to central limitation during effort are the impairment of pulmonary gas exchanges [24] direct myocardial injury, arrhythmias, and thromboembolism [25].

Therefore, what has been learned after several years of research on the modality of the adapted physical activity for the improvement of the $\dot{\mathrm{V}}{\mathrm{O}}_{2\mathrm{max}}$ in cardio-pulmonary disease is expected to be suitable and successful also in this new unexplored population of subjects recovering from the COVID-19 [26].

### Countermeasures

Among the vast number of the successful adapted physical activity that positively ameliorate the $\dot{\mathrm{V}}{\mathrm{O}}_{2\mathrm{max}}$ in the population suffering from central limitations (lungs and heart) high intensity interval training (HIIT) is becoming one of the most popular fitness programs, also because of its greater enjoyment with respect to standard endurance training [27]. Besides the potential appeal of HIIT, it is important to note that the physiological mechanisms activated by HIIT are different to those involved in endurance training in terms of central hemodynamics stimulation [28]. The limited ventilation, heart rate and cardiac output responses are indeed counterbalanced by high stimulation of the peripheral circulation. Therefore, also in the subjects recovering from the COVID-19 an adapted physical activity based on the HIIT approach will guarantee high-intensity muscular stimulus reducing cardio-pulmonary involvement. This approach could be very useful during the first period after hospital discharge when generally persist varies degrees of respiratory function impairment and in some cases a critical myopathy and neuropathy [29]. Another successful exercise modality used in patients with COPD and HF is small muscle mass exercise [30–32] characterized by an endurance training program performed at high intensity using limited muscles together (single-leg exercise). It has been described as a powerful approach to decrease exercise intolerance in patients with HF despite central hemodynamic limitations, allowing for a clear improvement in muscle structure and peripheral convective and diffusive oxygen transport, and subsequently the utilization of O_2_ [32]. These mechanistic findings may have important practical consequences even in patients with COVID-19 and should have to be evaluated. Nevertheless, clinical, and extensive application could be difficult because of the need for dedicated equipment [33].

Table 2 describes a proposal for an endurance training program for patients recovering from COVID-19 infection, based on more validated approaches evaluated in cardio-respiratory patients [19, 34]. Future studies on COVID-19 patients after the acute phase are warned to define specific training response characteristics and healing recovery trajectories. Baseline evaluation with the aim to assess the safety of the intervention and to better define the “dose” of exercise is highly suggested [18]. This should include the evaluation of physiological limitations by measuring lung function impairment, exercise tolerance, muscle function and balance, as well as patient-reported measures of Health-Related Quality of Life. Moreover, strictly monitoring of symptoms, oxygen saturation and heart rate during each exercise session are recommended [35].

## Skeletal muscle and COVID-19 consequences: resistance training as prevention and recovery

It is well known since the early 20s by Cuthbertson [36] that inactivity affects negatively the muscular system also in healthy subjects with a loss of nitrogen, phosphorous and calcium due to the non-use of muscles and bones. Forty years later, it was demonstrated that a medium-term bed confinement (20 days) leads to a loss of 28% of maximum oxygen uptake capacity and 11% of heart volume in young healthy individuals [37]. In post COVID-19 patients, the skeletal muscle loss is due not only merely to inactivity but also to the so-called “cytokine storm” [38, 39]. Skeletal muscle mass is the result of a dynamic equilibrium between protein synthesis (MPS) and protein breakdown (MPB). This equilibrium has many more interweaving than it seems: for example, amino acids derived from MPB represent 80% of the daily synthetized protein whilst only 20% of the new protein derive from diet protein intake [40]. In general, nutrients intake, hormones, and activity (considered as muscle contraction) influence the balance between MPS and MPB hence the whole muscle protein balance [41]. At basal fasting conditions the muscle protein balance is negative because MPB is higher (≅ 30%) than MPS [42]. Hence, during bed confinement as during COVID-19 there are many factors that can affect protein net balance (NB):

1. *Nutrition*: during COVID-19, even though patients are not under oxygen therapy or in Intensive Unite Care, due to the olfactory and gustatory dysfunctions, that are a common clinical presentation of mild‐to‐moderate forms of COVID-19 [43], there could be a reduced caloric and macronutrients intake [44], especially protein. Indeed, the recommended protein intake during illness is 1.3–2 g kg body weight [45]. This amount is higher than the one recommended for healthy individuals because hospitalized patients often underwent an increase in gluconeogenesis and an increase in muscle catabolism due to inactivity and inflammation [46]. Thus, malnutrition is an important issue in COVID-19 patients [47] and may influence post-hospitalization recovery period.
2. *Inactivity*: the symptoms of COVID 19 (muscle weakness, hypoxemic respiratory failure, etc.) even without hospitalization or intensive care unit staying, force the patient to reduce physical activity. Moreover, hypoxemic respiratory failure and ARDS may lead to muscle weakness [48] and increased muscle loss [49]. Generally speaking, also a low symptomatic COVID-19 infection leads to a reduced physical activity that can impair muscle mass and function [16].
3. *Inflammation*: inflammation acts negatively on protein net balance and SARS-CoV-2 trigger the so-called cytokine storm. It is well established that the aberrant release of pro-inflammatory cytokines and chemokines, induced by SARS-CoV-2 infection, is one of the main causes of fatal outcomes of COVID-19 syndrome [39]. A severe progression of COVID-19 disease is determined by a tardive interferon-gamma response with a prolonged inflammatory state and lower Treg, NK, and both CD4 + and CD8 + cells count [50, 51]. Moreover, a prolonged bed rest causes an elevated inflammatory burden and inflammation lowers MPS and increases MPB [52–55].

### Countermeasures

Resistance exercise training (RET) is the one greatest strategy to counteract skeletal muscle dysfunction [56]. RET can be performed with different modalities, for example, changing number of repetitions, number of sets, rest time, speed of movement and modifying the load in the eccentric face [57–61]. Traditionally, it is recommended to perform heavy-load contractions (i.e. 70–85% 1RM) and related medium–low number of repetitions (6–12 reps) to optimize skeletal muscle hypertrophy, whilst higher load and relative lower repetitions are suggested to implement muscle strength [59]. Even though many metabolic mechanisms were adduced to support the superiority of medium–low number of repetitions [62], recent findings suggest that low load (thus high repetition number) could be, at least, efficient as the highest load for muscle hypertrophy [41, 63, 64]. These data suggest that heavy load/high-intensity training is not mandatory to elicit an increase in muscle protein synthesis which is a prerequisite for skeletal muscle hypertrophy. Thus, in a COVID-19 patient, during the recovery period RET should be fundamental to gain skeletal muscle mass. Considering the complexity of the RET variables [65] a general scheme of RET progression may be suggested:

1. Start with three/four compound exercises with light loads and medium/high number of repetitions (15–18), with slow movement to increase the time under tension [63, 66] and, if possible, reach momentary failure [67].
2. Add single-joint exercise to increase volume [68].
3. Decrease repetitions and increase loads and rest between sets in compound exercises [69].
4. After 6/8 weeks a high-intensity interval resistance training may be proposed [70].

Finally, it is mandatory, during a RET schedule, finalized to an increase muscle mass, to pay attention to the diet to:

- Optimize hypertrophy mechanisms: 1.3–2 g of good quality protein (fish, poultry, lean meat, eggs) for kg of body weight.
- Reduce inflammation: increase the consumption of fresh vegetables and reduce the intake of refined sugar.

RET may be a fundamental tool to restore skeletal muscle mass and function in COVID-19 patients during the recovery period.

## The role of adapted physical activity to promote the mental health and psychological well-being of patients recovering from COVID-19 infection

The worldwide spread of the SARS-CoV-2 infection has forced governments of different Countries to adopt extreme measures including lockdown policies for all citizens except for essential services. Due to the emergency caused by the pandemic, huge efforts have been made to produce COVID-19 vaccines, the first of which were released in December 2020.

However, the spread of the vaccination among the population in general occurred only during the second quarter of 2021. During the previous months, the imposed quarantine due to the COVID-19 pandemic forced a drastic reduction in normal daily activities, by causing inactive lifestyles. The reduction of physical activity for healthy subjects and athletes triggered inactivity-associated disorders such as a decline in maximal oxygen consumption, reduction of endurance capacity, loss of muscle strength and mass, overweight, decrease joint lubrification [71–73].

Accumulated evidence has shown that physical activity is relevant to all healthy adults and represents an effective therapy for most chronic diseases by counteracting inflammation, muscle atrophy, degeneration of bone and cartilage, and aerobic capacity decrease [74, 75]. Regular physical exercise enhances behavioral performance, memory, executive functions, brain plasticity, neurogenesis and reduces the symptoms of neurodegenerative diseases [76, 77]. These beneficial effects of physical activity are noteworthy, considering that different neurological disturbances were encountered in COVID-19 affected patients, such as acute demyelinating encephalomyelitis or Guillain-Barré syndrome [78, 79].

The WHO has emphasized that physical activity is an important tool to counteract psychological distress and preserve the psychological well-being of individuals against the negative impacts of the pandemic (WHO, 2020). Physical exercise reduces depressive and anxiety symptoms, by improving one's self-esteem and a sense of well-being [80, 81]. The positive potential influence of physical activity is due to the involvement of the endogenous opioid system as well as the hypothalamic–pituitary–adrenal (HPA) axis, both implicated in anxiety, stress, depression, and emotional responses [82, 83]. Moreover, the positive role against anxiety and depressive disorders promoted by exercise is also due to the increased expression and release of neurotrophic and growth factors, including brain-derived neurotrophic factor (BDNF) and nerve growth factor (NGF), which are considered key modulators of psychological well-being [84].

### Countermeasures

The extant literature has suggested that regular physical activity may have an important role in psychological well-being in the unprecedented and potentially distressing context of COVID-19 lockdown [85]. Jenkins et al. [86] showed that weekly physical exercise was associated with increased psychological well-being during the COVID-19 lockdown, and the nature-based physical activity may strengthen the psychological well-being via effects on motivation. Another study performed across four Western nations (USA, UK, France, and Australia) demonstrated the deleterious effects of lockdown on the reduction of physical activity and mental health of subjects [87]. Lesser and Nienhuis [88], showed that physically active subjects reported greater mental health scores as compared to inactive subjects during the COVID-19 pandemic. Furthermore, the dramatic effects of negative impact on the psychological health and well-being of the population seem to be higher in females and young adult people [89].

The COVID-19 affected patients, during the acute phase, are obliged to rest in bed and are not able to make normal daily life activities, neither regular physical exercise [89].

However, in light of the several beneficial effects of physical activity, adapted exercise reeducation strategies are needed for a fast recovery of the patients and to ensure their psychological well-being.

Although most patients present mild forms of COVID-19, nearly 14% of subjects present severe forms, requiring hospitalization and oxygen therapy, and 5% need admission to intensive care units [90]. Due to the intensive medical management for some hospital-admitted COVID-19 patients, in the acute phase, it is possible to adopt only passive strategies performed by different health professionals, especially physiotherapists and/or kinesiologists, such as whole-body vibration exercise and passive range of motion [91]. In this scenario, typical passive mobilization training, such as passive stretching, should be adopted [92–95]. After the acute phase of respiratory distress to reduce the severity of intensive care unit-acquired weakness and induce rapid functional recovery, the physiotherapists and/or kinesiologists can plan a bed-based exercise program focusing on respiratory rehabilitation training, and chest relaxation training. Moreover, they have to assist patients to mobilize independently to stand and exert normal daily functions according to the Barthel Index, such as eating, walking, and so on. The early mobilization and an adapted physical activity plan are beneficial for individuals with COVID-19 also for the known effects on general well-being since these activities keep the patients busy who usually suffers from depression due to forced immobility. Rapid regaining mobility allows the patient to acquire better self-esteem and represents a strong response to depression, anxiety, and panic attacks. Weeks after being discharged from the hospital, the patient must gradually restart the physical activity program administered by a sport scientist to return to pre-infection fitness. In this phase, a tailor-made home-based exercise program decreases stress hormones, distracts the patient from negative thoughts and emotions, promotes confidence, and improves better mental health, ensuring faster recovery and a return to normal life.

**Fig. 1:**
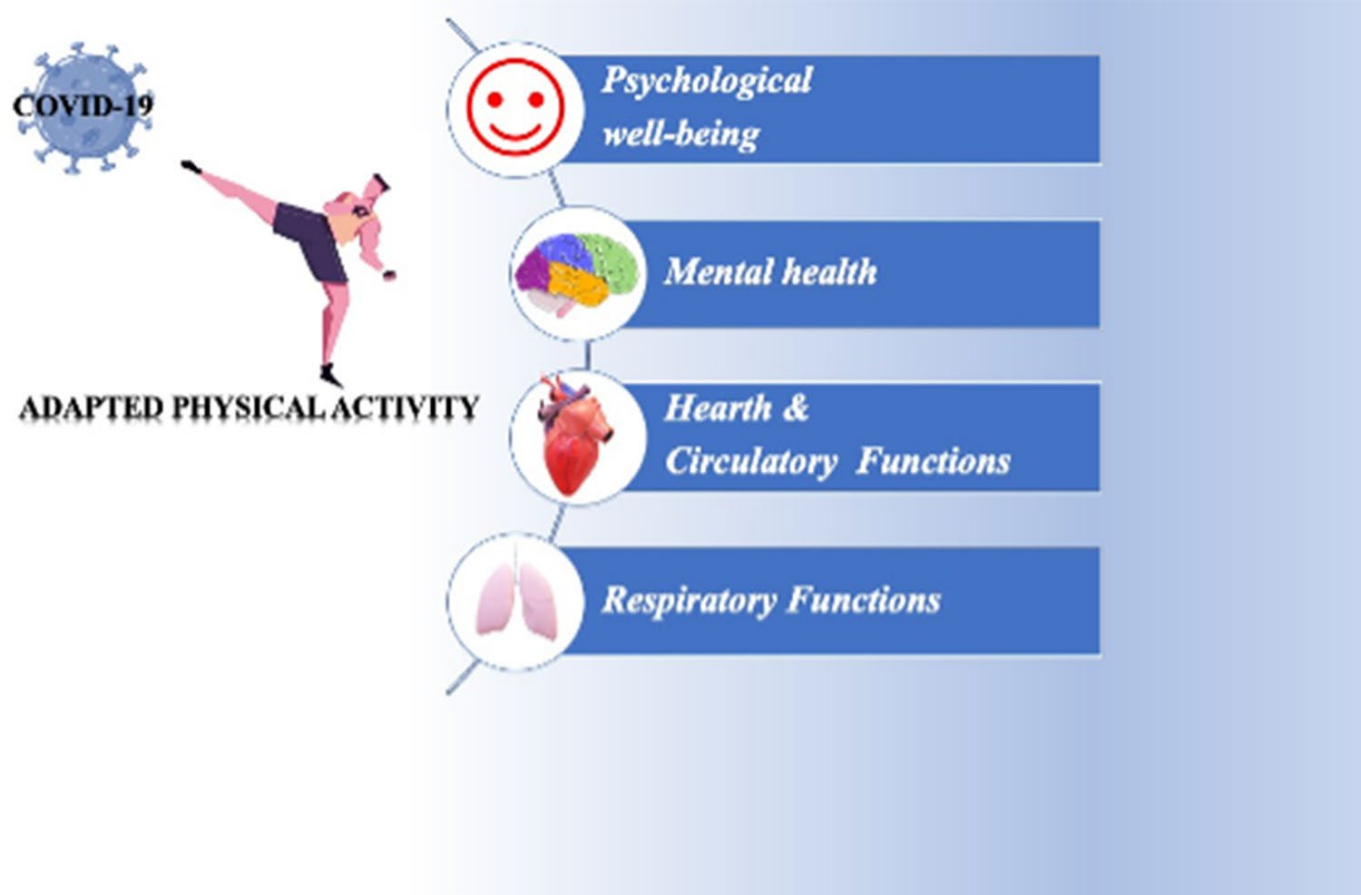
The beneficial effects of adapted physical activity to improve the physical and mental health in COVID-19-affected subjects

Considering that physical activity increases psychological well-being and outdoor exercise showed even more positive effects, moderate outdoor sports activities (e.g., jogging, Nordic walking, going for a walk) are strongly recommended, to allow the full recovery of patients. Nordic walking involves muscles of the upper body more than standard walking, since it requires the use of a specific pair of poles, by increasing the energy expenditure [96]. In conclusion, the adapted physical activity plan to COVID-19 affected patients is essential to prevent, reduce and rehabilitate the consequences of the disease as well as improve mental and psychological well-being (Fig. 1).

## Elite athletes’ management in era COVID-19

The COVID-19 outbreak caused, beside the danger to get sick, home confinement as well as the suspension of training and sport competitions. The disease is an illness with unknown causes and with a high viral load. The non-specific symptoms at early stages, especially in young and healthy people, such as athletes, lead to a serious danger of contagion [97].

From about 18 months after the start of the emergency, several specific issues regarding the sport activity resumption and elite athletes’ management are pertinent, including questions about the potential transmission of disease within teams, decisions on how to continue to train and the effect of vigorous exercise on infection susceptibility. The need for guidance, regarding return to train following COVID-19 infection, become urgent and different for any sport situation. Particularly athletes in Era-COVID-19 experienced only mild symptoms whether infected. However, being often potential asymptomatic vectors of the virus, they may contribute to the transmission of COVID-19 within their community, also within older or immunocompromised individuals.

Prevention of COVID-19 in athletes is also essential to minimize training interruptions, which lead to poorer performance [98]. The training interruptions reduce the total training load while the high intensity or volume of the training lead to a temporary immune system depression [99]. In fact, regular and moderate physical activity is associated with an improvement in the immune function, optimizing the body’s defence mechanisms against infections [100], conversely, high-intensity training, have been associated with transient immune perturbations, inflammation and oxidative stress, especially in recreational athletes [101]. Nevertheless, the conservative approach, linked to the application of COVID-19 prevention rules, may delay the athletes’ preparedness and event results [102].

Despite these considerations, the first and main goal of a well-planned training protocol for athletes must allow a safe sport activity resumption, investing in athlete health protection. The second aim is to mitigate, in social cooperation, the spread of COVID-19 carried by the asymptomatic athletes to the broader communities that include other athletes and their support teams [103].

### Countermeasures

To provide a safe return to training and competitions, in the fight against the COVID-19 transmission, the vaccination seems to be the most efficient strategy [104].

Moreover, to avoid the virus transmission during training, which is the second aim of a safe sport training resumption, it is needed to apply the universal COVID-19 guidelines such as social distancing, make use only of personal equipment, maintain personal hygiene, good nutrition, and almost 7 h of sleep per night, to correctly recover an athlete. The impact of sleep and recovery and on optimum readiness must be considered in this context [105].

For athletes with confirmed COVID-19, since in-home isolation, low-intensity indoor training, focused on maintenance of cardiovascular and strength endurance, may be attempted during that time. Guidance and monitoring by a strength and conditioning coach or exercise physiologist can be provided remotely. This conditioning program aims to maintain an acceptable training level and avoid psychological distress. Physical activity is well recognized, such as a good strategy for nurturing resilience. Elite Athletes demonstrated a high level of psychological distress reactions to their sport activity break [106]. Nevertheless, if adequately supported by social connections, including their teammates and coaches and their sport environment, they can successfully address the distress condition [100].

After discontinuing in-home isolation, an athlete can gradually return to training as tolerated. Training can begin once no symptoms are evident and energy levels return to normal [107], after a further mandatory medical examination, in addition to the usual certificate attesting suitability for competitive sporting activities, to exclude subclinical disease. A practical and recommended test evaluating the individual’s ability to jog for 10 min without changing the rhythm during the 10 min interval, could be used to allow the return to low to moderate training.

Teammates, coaches, and other staff who have had close contact with him/her should stay in-home isolation for 14 days. During this time, any symptoms experienced by other athletes or staff should be reported to the team physician to determine whether they are legitimate signs of COVID-19. Team physicians may also consider implementing daily temperature checks.

Best practices to allow a safe training management in the era of COVID-19 may be resumed as follows:

- guarantee a safe environment with sufficient spaces for each athlete’s training and an optimal ventilation for indoor sports;
- suspension of non-vaccinated athletes or Frequent Sars-COV2-PCR testing every 72/96 h for both vaccinated and no-vaccinated athletes [108];
- limiting the training session in term of total duration while incrementing the frequency sessions of the training sessions to guarantee the optimal intensity and rest, avoiding the temporary immune system depression;
- considering that the use of the face mask may interfere with normal ventilation, especially for elite athletes, a real-time monitoring of social distancing could safeguard against virus transmission.

To avoid athletes and coaches’ psychological discomforts, the new rules of the sport organization, in the era of COVID-19, have to be interiorized as essential for their health safety and training and career outgrowth and not perceived as compulsory or as their freedom loss. In this way, it could be possible to restart without risks [109].
